# Use of medical face masks versus particulate respirators as a component of personal protective equipment for health care workers in the context of the COVID-19 pandemic

**DOI:** 10.1186/s13756-020-00779-6

**Published:** 2020-08-06

**Authors:** John Conly, W. H. Seto, Didier Pittet, Alison Holmes, May Chu, Paul R. Hunter, on behalf of the WHO Infection Prevention and Control Expert Group for COVID-19, John Conly, Barry Cookson, Didier Pittet, Alison Holmes, May Chu, Andreas Voss, Anna Sara Shafferman Levin, Wing Hong Seto, Marimuthu Kalisvar, Dale Fisher, Nina Gobat, Paul R. Hunter, Mark Sobsey, Mitchell J. Schwaber, Sara Tomczyk, Moi Lin Ling

**Affiliations:** 1 University of Calgary and Alberta Health Services, Calgary, Alberta Canada; 2 University of Hong Kong , Hong Kong, China; 3 University of East Anglia, Norwich, UK; 4 Hopitaux Universitaires de Genève, Geneva, Switzerland; 5 Imperial College, London, United Kingdom; 6 Colorado School of Public Health, Aurora, Colorado USA

**Keywords:** SARS-CoV-2, COVID-19, Droplet, Contact, Airborne, Infection prevention, Transmission, Medical mask, N95 respirator

## Abstract

Currently available evidence supports that the predominant route of human-to-human transmission of the SARS-CoV-2 is through respiratory droplets and/or contact routes. The report by the World Health Organization (WHO) Joint Mission on Coronavirus Disease 2019 (COVID-19) in China supports person-to-person droplet and fomite transmission during close unprotected contact with the vast majority of the investigated infection clusters occurring within families, with a household secondary attack rate varying between 3 and 10%, a finding that is not consistent with airborne transmission. The reproduction number (R_0_) for the SARS-CoV-2 is estimated to be between 2.2–2.7, compatible with other respiratory viruses associated with a droplet/contact mode of transmission and very different than an airborne virus like measles with a R_0_ widely cited to be between 12 and 18. Based on the scientific evidence accumulated to date, our view is that SARS-CoV-2 is not spread by the airborne route  to  any significant extent and the use of particulate respirators offers no advantage over medical masks as a component of personal protective equipment for the routine care of patients with COVID-19 in the health care setting. Moreover, prolonged use of particulate respirators may result in unintended harms. In conjunction with appropriate hand hygiene, personal protective equipment (PPE) used by health care workers caring for patients with COVID-19 must be used with attention to detail and precision of execution to prevent lapses in adherence and active failures in the donning and doffing of the PPE.

## Background

The mechanisms of transmission (airborne, droplet, contact, vector or common vehicle) for microorganisms supports a specific combination of barrier precautions chosen on the basis of a point-of-care risk assessment by the health care worker (HCW) [1, 2]. Any person who is in close contact (generally considered to be within 1 m) with someone who has respiratory symptoms (e.g., sneezing or coughing) is at risk of being exposed to potentially infective respiratory droplets. Moreover, droplet transmission may also produce fomites on any surface in the immediate environment around the infected person. Airborne transmission refers to the presence of microbes within droplet nuclei (generally considered to be particles < 5–10 μm in diameter), which result from the evaporation of larger droplets and/or exist within dust particles and may remain in the air for long periods of time and may be transmitted to others over longer distances such as the measles virus [2–4]. However, it is important to recognize that in the course of medical care, aerosols of particles generally considered to be < 5–10 μm may be generated in certain procedures considered to be “aerosol-generating medical procedures” (AGMP) and transmitted at limited distances beyond 1 m, which has been referred to as “opportunistic” airborne transmission and airborne precautions are appropriate for these settings [4]. Within the context of the general understanding of the routes of droplet and opportunistic airborne transmission, controversy exists about the relative contribution and importance of the routes of each of them related to specific viruses. For example a systematic review of the literature concluded that influenza virus transmission in humans occurs only over short distances consistent with predominantly the droplet route [5], but Tellier suggested that limited aerosol transmission over longer distances can occur in addition to droplet transmission [6, 7]. It is recognized that there is a continuum of transmission routes between large droplet and aerosol and it is an important concept. Particles of a variety of sizes are expelled from the human airway during coughing, sneezing, talking and medical procedures.

The aerobiology of expired large droplets and smaller particles and the transmission dynamics to allow for a replication competen and tinfection competent virus to establish an invasive infection in humans is complex. The size of the particles and the distance the particles may be expelled is variable and depends on many factors, including the size distribution of the particles, the propulsive force generated by the individual or the procedure, the relative humidity, evaporation level, settling velocity, direction and velocity of air flow, the number of air changes per hour, temperature, crowding and other environmental factors. In addition there is variability in the type of the respiratory virus in question, the dispersion, quantity, and distribution of the virus within the droplets and smaller particles, the stability of the virus, its replication and infection competence, ability to enter the respiratory tract, ability to bind to specific host cell receptors and to establish invasive infection in a susceptible host. The process is further complicated by debate regarding how well the use of quantitative polymerase chain reaction (PCR) techniques performed on respiratory specimens can be interpreted with respect to recovery of viable virus and its titer, depending on the timing of presentation and stage of illness [8–12]. Regardless of the uncertainties, one certainty is that the use of Personal Protective Equipment (PPE) including gloves, gowns, medical masks and eye protection in combination with patient placement in adequately ventilated single rooms represents one component of the Infection Prevention and Control (IPC) response to prevent transmission of pathogenic microorganisms to HCWs [1, 2]. However the effectiveness of PPE depends on its availability, the proper physical environmental controls, adequate staff training, strict adherence to hand hygiene and appropriate human behaviour [13, 14].

## Modes of transmission of SARS-CoV-2

Currently available evidence supports that the predominant route of human-to-human transmission of the SARS-CoV-2 is through respiratory droplets and/or contact routes [1, 15–19]. The report by the World Health Organization (WHO) Joint Mission on Coronavirus Disease 2019 (COVID-19) in China which analyzed the experience with 75,465 cases supports person-to-person droplet and fomite transmission during close unprotected contact, with the majority of SARS-CoV-2 transmission occurring within families in close contact with each other [16]. The vast majority (78–85%) of the investigated infection clusters occurred within families, with a household secondary attack rate varying between 3 and 10%, a finding that is not consistent with airborne transmission [16]. The reproduction number (R_0_) for the SARS-CoV-2 was estimated to be between 2.0–2.5, compatible with influenza and other respiratory viruses typical for a droplet/contact mode of transmission and very different than a classical airborne virus such as measles which is estimated to have a R_0_ of greater than 10 and widely cited to be between 12 and 18 [16, 20]. Other detailed reports have also been consistent, finding a R_0_ of 2.2–2.7 for SARS-CoV-2 [21, 22].

Multiple clinical and epidemiologic reports have now lent considerable support that the predominant route of human-to-human transmission of the SARS-CoV-2 is through respiratory droplets and/or contact routes and do not support significant airborne transmission. An investigation of 25 close contacts sitting within 2 m of a symptomatic index case with cough and a presymptomatic case, both confirmed to have COVID-19, multiple exposed flight crew members and potentially all 350 passengers on board an airplane during a 15-h flight revealed no evidence of transmission of SARS-CoV-2 [19] supporting a droplet as opposed to airborne transmission route. Although the cases were reported to be wearing masks on the flight, it is not possible to wear masks during eating and drinking and the filtration capacity of the mask would not likely have been adequate for the entire 15 hour flight. Another report in a clinical setting in which 41 health care workers (HCWs) were exposed for over 10 min and within 2 m of a patient with confirmed COVID-19 during an intense and difficult intubation and non-invasive ventilation scenario, involving multiple AGMPs, revealed no transmission events of SARS-CoV-2 with repetitive testing of all the HCWs [23]. The majority (85%) of the HCWs were wearing a medical mask and other appropriate PPE while the remainder wore an N95 respirator. Another recent investigation of an initially undiagnosed COVID-19 patient with severe pneumonia with a confirmed high frequency of coughing and receiving oxygen therapy at 8 L/min who was nursed in an open 10 bed cubicle of a general ward for 35 h, with minimal spacing between patients, led to an exposure of 71 staff and 49 patients, including 7 staff and 10 patients who fulfilled the criteria of ‘close contact’ (within two metres of the index case for a > 15 min or had performed AGMPs without a N95 respirator), identified no SARS-CoV-2 nosocomial transmission events [24]. All patients and 6/7 staff with close contact tested negative for COVID-19 despite inconsistent use of medical masks by the patients and either use of medical masks or N95 respirators by the HCWs. In total 76 tests were performed on 52 contacts of which all were negative, and all other identified contacts remained asymptomatic during the 28 day post-contact surveillance period [24]. The authors concluded that SARS-CoV-2 is not spread by the airborne route and that basic infection control measures, including the use of medical masks, hand and environmental hygiene are adequate to prevent nosocomial transmission of SARS-CoV-2. Another recent study of 48 persons involved in a nosocomial outbreak of SARS-CoV-2 infections in the pediatric dialysis unit of the University Hospital of Münster found that after contact with the index case, 7 HCWs, 3 patients and one accompanying person became infected. All had either cumulative 15 min of face-to-face contact or were HCWs with exposure within a distance of ≤ 2 m, which occurred without use of any PPE. Of the remaining contacts who had shared the same indoor environment without face-to-face contact or who had contact but at a distance of > 2 m but without any use of PPE, none were found to be positive for COVID-19 on testing [25]. Additional data supporting that airborne transmission is not a predominant mode of transmission and therefore that N95 respirators or their equivalent are not required for routine use is accruing from sites which use only medical masks as the component of PPE in the care of COVID-19 patients but have a well-trained and prepared staff complement. There have been an estimated 5544 person hours of continuous HCW exposure to 132 COVID-19 inpatients, using PPE consisting of gowns, gloves, medical masks, and face shields or goggles for routine care and the addition of a N95 respirator for any AGMPs within “designated” COVID-19 medical wards at 4 acute care hospitals in Calgary, Canada over the first 2 months of care delivery with no nosocomial SARS-CoV-2 transmission events documented in any HCWs to date [26].

Data from studies that sampled surfaces in the environment for the presence of SARS-CoV-2 RNA in the immediate airspace surrounding infected patients who had known significant viral loads in their respiratory secretions have provided both negative and positive results [17, 18, 27–30]. Several studies have now reported positive results for the presence of SARS-CoV-2 RNA in air samples but in extremely low copy numbers/m^3^ or per liter of air sampled and would be highly unlikely to represent viable virus [28–30]. No studies to date have been able to find viable SARS-CoV-2 within air samples [30]. Even if viable virus were to be found in air samples, it would need to be demonstrated that SARS-CoV-2 in the samples was both replication and infection competent in the context of health care settings where PPE is being used appropriately in conjunction with diligent hand hygiene to consider that airborne transmission represents a significant mode of transmission.

A recent experimental laboratory study suggested that aerosol transmission of SARS-CoV-2 is plausible, because they demonstrated that the virus can remain viable in aerosols for 3 h based on their experimental design. However, they used a Collison 3-jet nebulizer to shear a large volume liquid suspension of a high viral inoculum to generate aerosolized viral particles which were then impacted against a hard surface inside a drum [31]. This mode of artificial mechanical aerosol production has been used for testing bioterrorism agents [32, 33] and has little relevance to a coughing patient with COVID-19 in the clinical setting and does not offer evidence that the virus is routinely present in aerosols at the bedside. Another report suggested that based on laser light scattering observations, loud speech could emit oral droplet nuclei of about 4um in size that persist as a slowly descending cloud which remain airborne for more than 8 min and theoretically could contain viable virus capable of being inhaled into the lungs [34]. However this conjecture is dependent on the independent action hypothesis (IAH) and the authors readily admit that there is no evidence the IAH is valid for humans and SARS-CoV-2.Other reports have suggested that airborne transmission is a significant route of transmission for the SARS-CoV-2; the title of one report suggests that the world should face the reality that the virus is airborne [35–37]. These studies represent opinion pieces, one systematic review of mainly modelling plus some experimental studies, and brief case reports which do not utilize robust methods to rule out contact or fomite transmission or opportunistic airborne transmission.

A recent WHO report indicated that SARS-CoV-2 RNA has been detected in in feces in 30% of cases within a few days of symptom onset and live virus was cultivated from stools in some cases [16]. This latter observation and our knowledge of the extensive transmission that has emerged in hundreds of outbreaks of norovirus on cruise ships raises the possibility of the fecal-oral route as an additional means of transmission for SARS-CoV-2 which deserves attention and further study [38–40]. A recent report from the Diamond Princess cruise ship reported that before disinfection, SARS-CoV-2 RNA was identified on multiple surfaces up to 17 days after cabins were vacated from both symptomatic and asymptomatic infected passengers suggesting widespread contamination but likely no viable virus was present [41]. Similar extensive environmental contamination of surfaces by SARS-CoV-2 from infected patients has been reported [18]. Additional evidence is emerging about the recognition of contact as a major route of transmission with a recent report from China finding poor hand hygiene before and after contact with patients and improper PPE as significantly associated with HCW with poor hand hygiene being retained in the logistic regression with the highest relative risk [42].

## The choice of masks as a component of personal protective equipment for health care workers - what is the evidence?

Guidance from the WHO states that “health care workers should wear a medical face mask (herein after termed medical mask) when entering a room where patients suspected or confirmed of being infected with SARS-CoV-2 are admitted and in any situation of care provided to a suspected or confirmed case”. The use of a particulate respirator at least as protective as a US National Institute for Occupational Safety and Health (NIOSH)-certified N95, European Union (EU) standard FFP2, or equivalent, is recommended when performing aerosol-generating medical procedures [1, 2]. Some jurisdictions and professional societies have suggested that the precautionary principle [43] should be applied in the event of an outbreak of any new respiratory virus. In the context of the current COVID-19 outbreak, several institutions initially issued guidance indicating that particulate respirators (designed to protect against 95% of airborne particulates when tested against a 0.3-μm particles) should be used as a component of the PPE for the HCWs, rather than medical masks. Persisting with this approach and the subsequent differences in recommendations for the type of masks creates risk perception disparities for HCWs, which may be increased in jurisdictions in the world with limited or no access to particulate respirators, and in the event of domestic or global supply disruptions.

Strict adherence to the use of administrative controls and using medical masks as a component of PPE were shown to be effective with no reported transmission events to HCWs during the SARS outbreak in 2003 [44, 45] and in one setting without the use of airborne isolation rooms [45]. Although the appropriate use of fit tested particulate respirators as a component of PPE may be equally effective compared the use of medical masks for HCWs in the management of patients infected with coronavirus strains including SARS-CoV-2, it is important to note that there were multiple reports documenting SARS coronavirus transmission to HCWs despite the use of particulate respirators in conjunction with other PPE in accordance with guidelines which reflect failures to prevent transmission to HCWs using them [46–52]. The mechanisms of transmission in these latter settings are not well understood but draw attention to the points that the use of particulate respirators as a component of PPE do not provide infallible levels of protection to HCWs. It is likely that these failures relate to inappropriate use or self contamination events. Multiple studies using systems-based human factors analysis have demonstrated that lapses in adherence and active failures in the donning and doffing of PPE resulting in self-contamination, which may be the genesis of inoculation events leading to transmission of pathogens to HCWs [53, 54]. A review of the literature following the SARS outbreak in 2003 suggested that for PPE to be effective, its use should be as uncomplicated as possible and focus on key principles, strict adherence to protocols including those related to appropriate use of PPE, high compliance, and lend itself to achieve the highest level of effectiveness in preventing HCW transmission events [55, 56]. These studies suggest there is a need to simplify the PPE processes to ensure that compliance may be achieved.

There were multiple reports of SARS among HCWs in hospital outbreaks reported from Canada, China, Hong Kong, Taiwan and Vietnam followed by MERS outbreaks with HCW transmission events in the Middle East and South Korea which were caused by a very similar coronavirus to the SARS-CoV-2. These hospital outbreaks serve to focus attention on the critical importance of IPC practices, including appropriate PPE use, and having adequate training and knowledge among HCWs to ensure that PPE, barrier precautions and hand hygiene practices are used appropriately [1, 2, 14]. The single most important concept identified in the management of patients affected by viruses transmitted by the droplet/contact route is the precision of execution in the use of PPE, and which should be the primary focus rather than on the type of mask used by HCWs as a component of PPE.

The findings from multiple systematic reviews and meta analyses over the last decade have not demonstrated any significant difference in the clinical effectiveness of particulate respirators compared to the use of medical masks when used by HCWs in multiple health care settings for the prevention of respiratory virus infections, including influenza [57–59]. A recent large well conducted cluster randomized multi-center, multi-year pragmatic effectiveness study study no evidence of greater clinical effectiveness of particulate respirators compared to medical masks in the prevention of acquisition of laboratory confirmed influenza in HCWs [60]. One of the systematic reviews commented about the harms of particulate respirators, especially when worn for prolonged periods [57]. Other studies have demonstrated side effects associated with the use of particulate respirators including facial dermatitis from the respirator components, increased work of breathing, respiratory fatigue, impaired work capacity, increased oxygen debt, early exhaustion at lighter workloads, elevated levels of CO_2_, increased nasal resistance, and increased non-compliance events leading to self-contamination (adjustments, respirator or face touches, under-the-respirator touches, and eye touches) [61–67]. These side effects are not encountered with the same frequency with the appropriate use of medical masks. An additional study has suggested pregnant women were not able to maintain their minute ventilation and had decreased oxygen uptake and increased carbon dioxide production even at rest [55]. The effects on the developing fetus are unknown. Studies of the use of particulate respirators in clinical settings have demonstrated anywhere between 44 and 97% of HCWs do not use the respirators properly [68].

## Conclusion

Our view is that the weight of the scientific evidence to date indicates that particulate respirators offer no advantage over medical masks as a component of PPE for the prevention of respiratory viral infections transmitted by the droplet/contact route, when used for routine care in clinical settings. To date, the available evidence supports that the predominant route of transmission of SARS-CoV-2 is consistent with the droplet/contact route. There are potential unintended consequences of the use of particulate respirators that put HCWs at risk particularly with prolonged use, which have not been associated with the use of medical masks. HCWs should be apprised accordingly in an open and transparent manner regarding potential harms of particulate respirators in jurisdictions where particulate respirators are chosen for routine use as a component of PPE. In addition, particulate respirators are more costly, require fit testing, necessitate additional time and resources, do not provide an adequate fit in individuals with beards, and may provide a false sense of security. Moreover, in the current COVID-19 pandemic, shortages have been documented from overuse such that respirators were not available in settings where AGMPs are performed and where is evidence for their need. Regardless of whether jurisdictions choose the precautionary principle with consequent use of particulate respirators instead of medical masks as a component of PPE for routine care of COVID-19 patients, this choice must not detract from the critical importance of emphasizing that PPE is only one measure within a bundle that comprises administrative, environmental and engineering controls, as described in WHO’s infection prevention and control of epidemic- and pandemic-prone acute respiratory infections in health care [2].

PPE used by HCWs caring for patients with COVID-19 must be used with attention to detail and precision of execution which involves selecting the proper PPE and being trained in how to correctly don, doff and dispose of it – without self-contaminating oneself in the process, the latter underscoring the importance and attention required for hand hygiene. Additional evidence on the use of medical masks and respirators needs to be generated to help define and inform knowledge gaps as we learn more about the COVID-19 epidemic and HCW practices.

## Data Availability

Not applicable.
