# Correction to: Use of medical face masks versus particulate respirators as a component of personal protective equipment for health care workers in the context of the COVID-19 pandemic

**DOI:** 10.1186/s13756-020-00810-w

**Published:** 2020-09-09

**Authors:** John Conly, W. H. Seto, Didier Pittet, Alison Holmes, May Chu, Paul R. Hunter, on behalf of the WHO Infection Prevention and Control Research and Development Expert Group for COVID-19

**Affiliations:** 1 University of Calgary and Alberta Health Services, Calgary, Alberta Canada; 2 University of Hong Kong, Hong Kong, China; 3 University of East Anglia, Norwich, UK; 4 Hopitaux Universitaires de Genève, Geneva, Switzerland; 5 Imperial College, London, United Kingdom; 6 Colorado School of Public Health, Aurora, Colorado USA

**Correction to: Antimicrob Resist Infect Control 9, 126 (2020)**

**https://doi.org/10.1186/s13756-020-00779-6**

After publication of this article [1], the author reported that the institutional author “Expert Group” name in both the authorship list and the Competing Interests section should be corrected to: ‘WHO Infection Prevention and Control Research and Development Expert Group for COVID-19’.
